# Inhibition of SARS-CoV-2 nucleocapsid protein–RNA interaction by guanosine oligomeric RNA

**DOI:** 10.1093/jb/mvad008

**Published:** 2023-02-07

**Authors:** Ryoya Sekine, Satsuki Tsuno, Hayato Irokawa, Kazuhiro Sumitomo, Tianxue Han, Yusuke Sato, Seiichi Nishizawa, Kouki Takeda, Shusuke Kuge

**Affiliations:** Division of Microbiology, Faculty of Pharmaceutical Sciences, Tohoku Medical and Pharmaceutical University, 4-4-1, Komatsuhima, Aoba-ku, Sendai, Miyagi 981-8558, Japan; Division of Microbiology, Faculty of Pharmaceutical Sciences, Tohoku Medical and Pharmaceutical University, 4-4-1, Komatsuhima, Aoba-ku, Sendai, Miyagi 981-8558, Japan; Division of Microbiology, Faculty of Pharmaceutical Sciences, Tohoku Medical and Pharmaceutical University, 4-4-1, Komatsuhima, Aoba-ku, Sendai, Miyagi 981-8558, Japan; Division of Community Medicine, Faculty of Medicine, Tohoku Medical and Pharmaceutical University, 1-15-1, Fukumuro, Miyagino-ku, Sendai, Miyagi 983-8536, Japan; Department of Chemistry, Graduate School of Science, Tohoku University, 6-3, Azaaoba, Aoba-ku, Sendai, Miyagi 980-8578, Japan; Department of Chemistry, Graduate School of Science, Tohoku University, 6-3, Azaaoba, Aoba-ku, Sendai, Miyagi 980-8578, Japan; Department of Chemistry, Graduate School of Science, Tohoku University, 6-3, Azaaoba, Aoba-ku, Sendai, Miyagi 980-8578, Japan; Division of Microbiology, Faculty of Pharmaceutical Sciences, Tohoku Medical and Pharmaceutical University, 4-4-1, Komatsuhima, Aoba-ku, Sendai, Miyagi 981-8558, Japan; Division of Microbiology, Faculty of Pharmaceutical Sciences, Tohoku Medical and Pharmaceutical University, 4-4-1, Komatsuhima, Aoba-ku, Sendai, Miyagi 981-8558, Japan

**Keywords:** SARS-CoV-2, RNA < Viruses, RNA Interactions < Protein, N protein, liquid–liquid phase separation*Abbreviation:* COVID-19, Coronavirus Disease-2019; CTD, C-terminal domain; DIC, differential interference contrast; EMSA, electrophoretic mobility shift assay, IDR, intrinsically disordered region; FITC, fluorescein isothiocyanate; HEPES, 4-(2-hydroxyethyl)-1-piperazineethanesulfonic acid, LLPS, liquid–liquid phase separation; NTD, N-terminal domain; N, nucleocapsid; PEG, polyethylene glycol; PNA, peptide nucleic acid; SARS-CoV-2, severe acute respiratory syndrome coronavirus-2; SARS, severe acute respiratory syndrome coronavirus; vRNP, viral ribonucleoprotein

## Abstract

The interaction of the *β*-coronavirus severe acute respiratory syndrome coronavirus-2 (SARS-CoV-2) nucleocapsid (N) protein with genomic RNA is initiated by specific RNA regions and subsequently induces the formation of a continuous polymer with characteristic structural units for viral formation. We hypothesized that oligomeric RNAs, whose sequences are absent in the 29.9-kb genome sequence of SARS-CoV-2, might affect RNA–N protein interactions. We identified two such hexameric RNAs, In-1 (CCGGCG) and G6 (GGGGGG), and investigated their effects on the small filamentous/droplet-like structures (< a few μm) of N protein–genomic RNA formed by liquid–liquid phase separation. The small N protein structures were sequence-specifically enhanced by In-1, whereas G6 caused them to coalesce into large droplets. Moreover, we found that a guanosine 12-mer (G12, GGGGGGGGGGGG) expelled preexisting genomic RNA from the small N protein structures. The presence of G12 with the genomic RNA suppressed the formation of the small N protein structures, and alternatively apparently altered phase separation to induce the formation of large droplets with unclear phase boundaries. We showed that the N-terminal RNA-binding domain is required for the stability of the small N protein structures. Our results suggest that G12 may be a strong inhibitor of the RNA–N protein interaction.

## Graphical Abstract

**Graphical Abstract:**
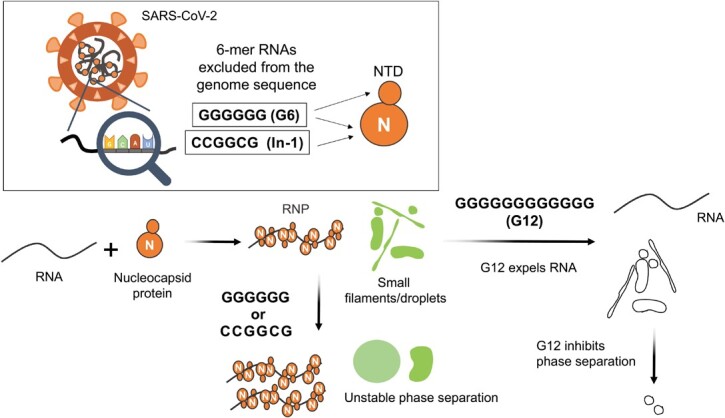

A novel type of severe acute respiratory syndrome (SARS) coronavirus (SARS-CoV-2) that causes severe acute pneumonia called COVID-19 (Coronavirus Disease-2019) emerged in Wuhan, China, at the end of 2019 *(**1**)*. The accumulation of SARS-CoV-2 genomic RNA mutations has led to host immune escape, resulting in the repeated spread of COVID-19. More than 636 million confirmed cases and a 1.0% death rate were reported in November 2022 (WHO COVID-19 dashboard; *(**2**)*).

The SARS-CoV-2 29.9-kb genome is a single-stranded positive-sense RNA encoding four structural proteins: the spike (S) protein, the envelope (E) protein, the membrane (M) protein and the nucleocapsid (N) protein *(**3**)*. The N protein is the most abundant viral protein in infected cells *(**4**)* and binds to genomic RNA to form an RNA–N protein complex (viral ribonucleoprotein [vRNP]) for packaging. The architecture of the RNA–N protein ultrastructure is likely based on protein–protein interactions and the interaction of the N protein and genomic RNA *(**5**)*.

The coronavirus N protein has two conserved domain structures consisting of the RNA-binding domain in the N-terminal domain (NTD) and a dimerization domain in the C-terminal domain (CTD) *(**6**,**7**)*. These domains are flanked by three intrinsically disordered regions (IDRs) located at the N-terminal end (N-IDR), in the middle (Linker-IDR) and at the C-terminal end (C-IDR) of the protein *(**8**)* (Fig. 1A). The structures of the three IDRs, NTD and CTD are conserved within *β*-coronaviruses *(**8**)* and are all predicted to bind RNA *(**9**,**10**)*. Cryo-electron tomography and subtomogram averaging analyses show that the CTD-mediated N protein dimer is L-shaped, with five dimers connected head-to-tail to form a G-shaped decamer structure (~15 nm in diameter) *(**5**)*. Multiple G-shaped decamers appear to form an ‘eggs-in-nest’ *(**5**)* or ‘beads on a string’ *(**11**)* shape in the virus particle and are thought to form in the presence of the viral genomic RNA *(**5**)*. However, the structure of the viral RNA and the G-shaped decameric complex remains unclear. In addition, it has not been clarified whether there is a preference for the nucleotide sequences that bind to each region and domain of the N protein.

**Fig. 1:**
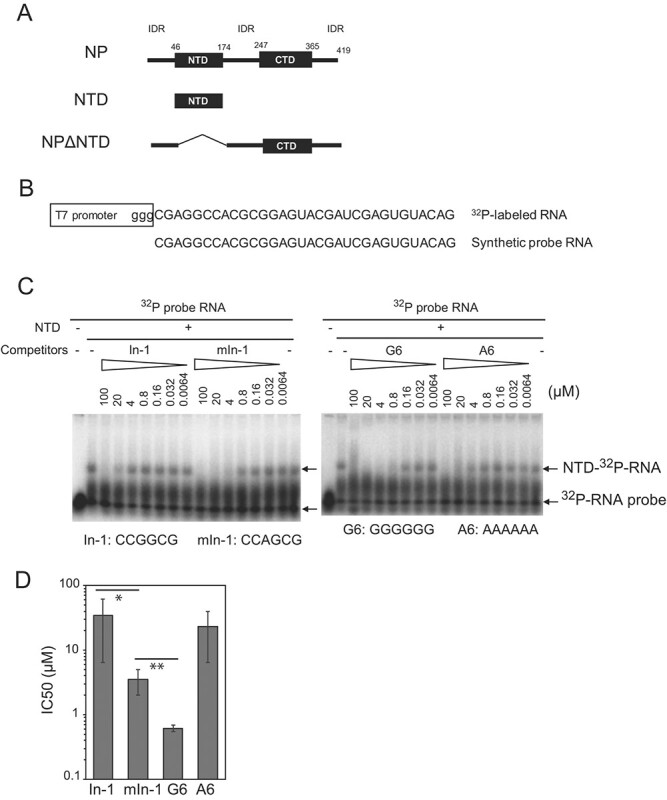
**Inhibition of the NTD–RNA interaction by hexameric RNAs absent from the SARS-CoV-2 genomic RNA sequence. A**. Structures of the N protein (NP), N-terminal RNA-binding domain (NTD) and NTD-deleted NP (NPΔNTD) are illustrated with the position of the domain. Intrinsically disordered regions (IDR) are shown. **B**. The probe RNA sequence (NC_045512.2, nt29733–29,764) is shown with the T7 promoter-driven nucleotide. Synthetic probe RNA was used for LLPS experiments. **C**. The inhibition of the NTD–RNA interaction by In-1, mIn-1, G6 and A6, determined by EMSA. The concentrations of the hexamers are indicated. Arrows indicate the positions of free probe RNA and RNA–NTD shifts. **D**. The IC_50_ of each hexamer was compared. The mean values and standard deviation (SD) of seven, six, four and three independent experiments are shown for In-1, mIn-1, G6 and A6, respectively. Variances were checked with F-tests, following which Student’s *t*-test was performed under the condition of unpaired equal variances (See Supplementary Fig. S1D). ^*^ and ^**^ indicate *P* = 0.03 and *P* = 0.01, respectively.

Many reports have shown that the N protein binds RNA, resulting in liquid–liquid phase separation (LLPS) *in vitro (**8**,**12–21**)*. The formation of droplets by the LLPS of N proteins in the presence of viral RNA is believed to be an *in vitro* model for vRNP formation *(**8**,**12**,**14**,**21**)*. LLPS, which occurs when N proteins and cellular RNA and proteins interact, is an intracellular suppressor of innate immunity *(**18**,**22**)*. Furthermore, the intracellular function of the N protein has been suggested to be regulated by its modification *(**12**)*: phosphorylation of the serine-arginine-rich region flanking the NTD induces viral RNA replication, while non-phosphorylated forms induce virus encapsulation *(**12**)*. The N protein has no RNA sequence specificity *(**9**,**10**)* and binds to the entire RNA sequence of 29.9 kb to form a polymer. Multiple RNAs and multiple N proteins bind and coalesce to form large droplets by LLPS *(**8**,**12–21**)*. However, one molecule of genomic RNA is thought to be bound to multiple N proteins and packaged into a virus particle *(**5**,**8**,**11**)*. A model has been proposed to explain how a single genome is selectively packaged, namely that symmetry breaking promotes single-polymer over multi-polymer assembly in the presence of a high-affinity site *(**8**)*. Possible initiation signals (ISs) for vRNP formation in the 5′ noncoding region and N protein coding region in the 3′ end of the SARS-CoV-2 genomic RNA were identified by the induction of droplet formation and N protein binding *in vitro* under physiological conditions *(**14**)*. The interaction consisting of N protein binding to ISs of the viral genome might drive the formation of the G-shaped vRNP complex to be packaged in a virus particle. On the other hand, the N protein itself may also be able to form N protein–RNA polymers in an ordered fashion *(**12**)* through the polymerization of N protein dimers into decamers by binding to RNA. Therefore, we considered that the phenomenon wherein droplets do not expand randomly is due to the intrinsic function of the N protein.

Based on the above findings, molecules that influence N protein-driven droplets could be targets for drug discovery to inhibit viral replication and vRNP formation *(**14**,**17**,**20**)*. Here, we investigated the effect of hexameric RNAs whose sequences are not present in the 29.9-kb RNA genome of SARS-CoV-2 on the NTD-binding activity and formation of LLPS droplets of the N protein induced by genomic RNA. We found that one hexamer with low binding affinity to the NTD induced the formation of large spherical droplets of the N protein. Another hexamer (guanosine oligomer G6) with a high binding affinity to the NTD could destabilize genomic RNA-induced phase separation. Our results also suggested that G12 might act as an inhibitor of the N protein–probe RNA interaction.

## Results

### Inhibition of the interaction between the NTD and probe RNA by hexameric RNA

We hypothesized that sequences that do not favor vRNP formation might have been eliminated during the evolution of SARS-CoV-2. We found that In-1 (CCGGCG) and G6 (GGGGGG) are absent in both the plus and minus strands of the RNA genome (Supplementary Fig. S1B). Therefore, we investigated the inhibitory activity of these hexamers using the electrophoretic mobility shift assay (EMSA), which detects NTD–RNA interactions. mIn-1-1 (CCAGCG), a control hexameric RNA with a single base substitution of In-1, and a homo-adenosine hexamer (A6; AAAAAA) were used as controls. A previous study *(**23**)* demonstrated using EMSA that the NTD of infectious bronchitis virus (IBV), an avian *β*-coronavirus, could bind to the 3′-noncoding region of the IBV genome. We found that a 32-base sequence in IBV’s 3′ noncoding region is highly conserved in several *β*-coronaviruses, including SARS-CoV-2 (Supplementary Fig. S1C). This 32-base sequence is not included among the ISs of SARS-CoV-2 *(**14**)*. Hence, we used this 32-base sequence as a probe RNA (Fig. 1B) for the EMSA of the SARS-CoV-2 NTD.

Interestingly, G6 showed the strongest inhibitory effect (IC_50_ = 0.62 ± 0.07μM), whereas A6 (IC_50_ = 22.9 ± 16.4 μM), In-1 (IC_50_ = 34.3 ± 27.8 μM) and mIn-1 (IC_50_ = 3.48 ± 1.46 μM), a G to A mutant of In-1, exhibited lower levels of inhibition (Fig. 1C and D). These hexameric RNAs might be monovalent binders of the NTD *(**21**)* and might act as competitive inhibitors of probe RNA binding to the NTD. These results suggest that these RNA oligomers inhibited the NTD–RNA interaction in a sequence-specific manner.

### Induction of N protein phase separation by probe RNA and hexameric RNAs

The SARS-CoV-2 N protein forms non-membranous droplets with RNA via LLPS *in vitro* (see above for references). To investigate the function of NTD as an RNA-binding domain on RNA-induced phase separation, we examined an NTD deletion mutant of the HIS-tag N protein (NPΔNTD) in addition to the HIS-tag full-length N protein (NP). We purified the proteins from *Escherichia coli* under denatured conditions and renatured them for use (Supplementary Fig. S2A).

**Fig. 2:**
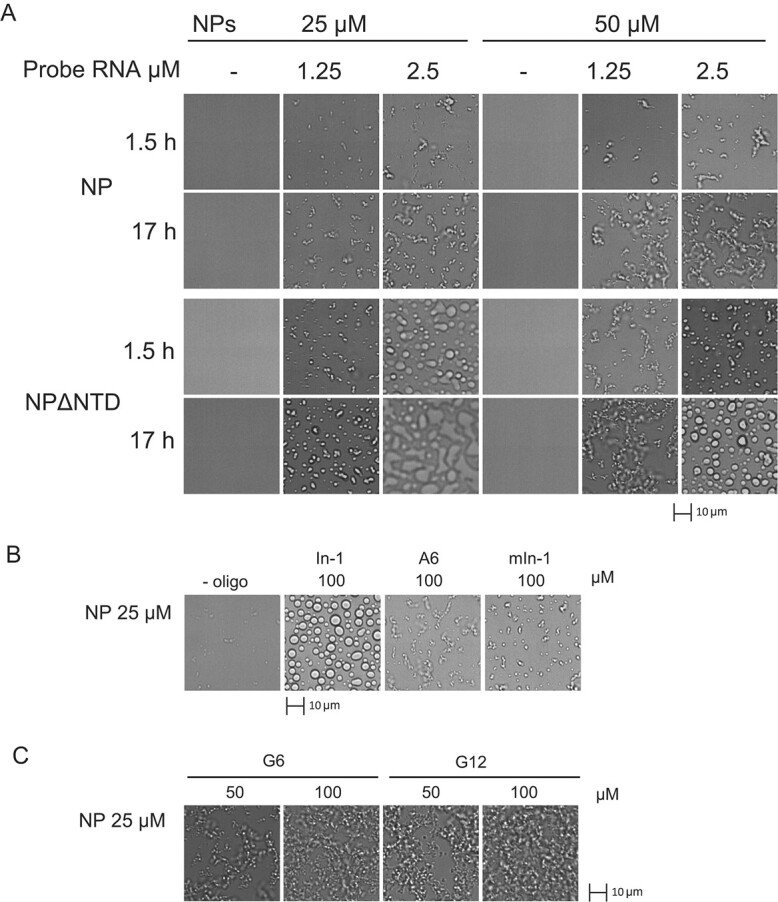
**Phase separation of NP and NPΔNTD induced by the probe RNA and hexameric oligo-RNA. A**. The 32-base RNA in the 3′ noncoding region of the SARS-CoV-2 genome was used as probe RNA. NP and NPΔNTD (25 and 50 μM) were mixed with 1.25 and 2.5 μM probe RNA or left without probe RNA (−) for 1.5 h and 17 h. DIC images were obtained. Scale bars of 10 μm are shown. Corresponding data for NTD are shown in Supplementary Fig. S2B. **B**. NP (25 μM) was mixed with 100 μM of hexameric oligo-RNA (A6, In-1 and mIn-1). **C**. G6 and G12 induced filamentous LLPS. NP (25 μM) was mixed with the indicated concentration of oligomeric RNA overnight.

We examined the appearance of droplets of NP and NPΔNTD, which were induced by probe RNA on glass surfaces coated with polyethylene glycol (PEG)-silane *(**24**)*, and compared them with differential interference contrast (DIC) microscopy at 27°C. Small filamentous/droplet-like structures were induced by incubation with probe RNA for 1.5 h (Fig. 2A), and the levels of these structures increased with further incubation (17 h, Fig. 2A). By contrast, the NTD that could bind to the probe RNA (Fig. 1C) failed to exhibit any of the structures (Supplementary Fig. S2B). The droplet of NPΔNTD coalesced at 1.5 h and underwent two-phase separation with a large area after 17 h (Fig. 2A) when the probe RNA levels were increased from a molar ratio of 0.05 to 0.1 (2.5 μM probe RNA/25 μM NPΔNTD). Unlike the phase change exhibited by NPΔNTD, NP formed small filamentous/droplet-like structures even with higher levels of probe RNA (Fig. 2A). These results suggested that the NTD can suppress the formation of large spherical droplets as a domain of the N protein.

Next, we examined whether the hexameric RNAs could induce phase separation. Interestingly, In-1 strongly induced the formation of large spherical droplets of NP, but mIn-1 and A6 induced low-level small filamentous/droplet-like structures of NP (Fig. 2B). G6 strongly induced small filamentous aggregates of NP at high levels (Fig. 2C). Because a hexamer act as a monovalent binder but not as a bridging effector *(**21**)*, In-1 and G6 might alter the N protein structure and induce interactions between N proteins.

### Inhibitory effect of oligo-RNAs on the formation of small filamentous/droplet-like structures of the N protein

**Fig. 3:**
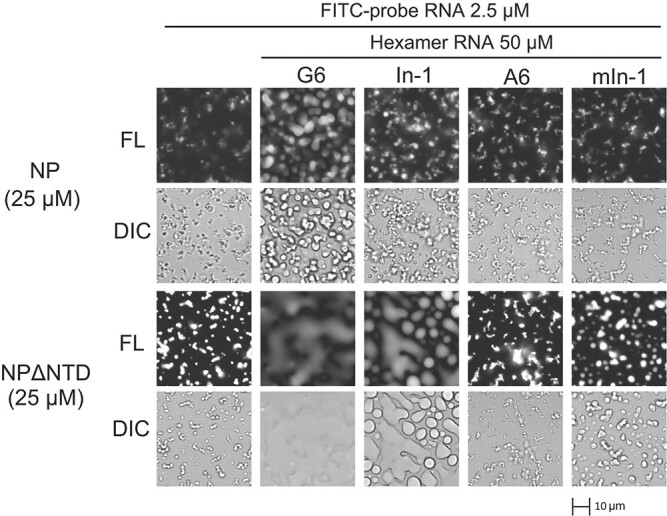
**Effect of hexameric RNAs on the probe RNA–induced structures of NP and NPΔNTD.** NP and NPΔNTD (25 μM each) were incubated with 2.5 μM of FITC-labeled 32-base probe RNA (FITC-probe RNA) for 3 h, followed by 50 μM of hexameric RNA (G6, A6, In-1 and mIn-1) for 16 h. DIC (DIC) and fluorescent (FL) images are shown. A scale bar of 10 μm is shown. Time- and concentration-dependent changes in the LLPS images (1/10 scale) and 25 μM and 50 μM hexameric RNA treatments, including the present data, are shown in Supplementary Fig. S3. Note that the fluidic phase changes of probe RNA–NPΔNTD (Fig. 2A) were weaker when FITC-probe RNA was used (compare Figs 2A and 3, 2.5 μM probe RNA versus 25 μM NPΔNTD).

We hypothesized that the N protein–RNA structures might be inhibited by the abovementioned hexameric RNAs through competition and displacement of the probe RNA or the allosteric effect of the hexameric RNA on the N protein. Thus, we monitored changes in the small filamentous/droplet-like structures using a fluorescently-labeled probe RNA (fluorescein isothiocyanate [FITC]-probe RNA; unlabeled and FITC-labeled probe RNA were mixed evenly). NP and NPΔNTD were incubated with FITC-probe RNA for 2 h before hexameric RNA was added. We monitored changes in phase separation using fluorescence and DIC microscopy (Fig. 3 and Supplementary Fig. S3). G6 enhanced the droplets due to LLPS, whereas In-1 enhanced the filamentous/droplet-like structures of NP in a concentration-dependent manner (Supplementary Fig. S3A). Moderate effects of A6 and mIn-1 on the small filamentous/droplet-like structures of both NP and NPΔNTD were observed (Fig. 3 and Supplementary Fig. S3A). Interestingly, G6 and In-1 altered the probe RNA-induced NPΔNTD phase separation in a hexamer concentration-dependent manner. Droplets formed by nucleation appeared to coalesce over time and became nearly uniform, with unclear phase boundaries (Supplementary Fig. S3 and Fig. 3, NPΔNTD). This was more remarkable when the concentration of RNA was high (Supplementary Fig. S3, 50 μM compared to 25 μM, NPΔNTD). For NP, G6 had a similar, albeit weaker, effect on the changes in phase separation (Fig. 3, NP). These results suggest that G6 and In-1 possess RNA sequences that alter the NP–probe-RNA interaction and phase separation state of NPΔNTD.

### Guanosine 12-mer RNA expels probe RNA from the N protein filamentous/droplet-like structures

**Fig. 4:**
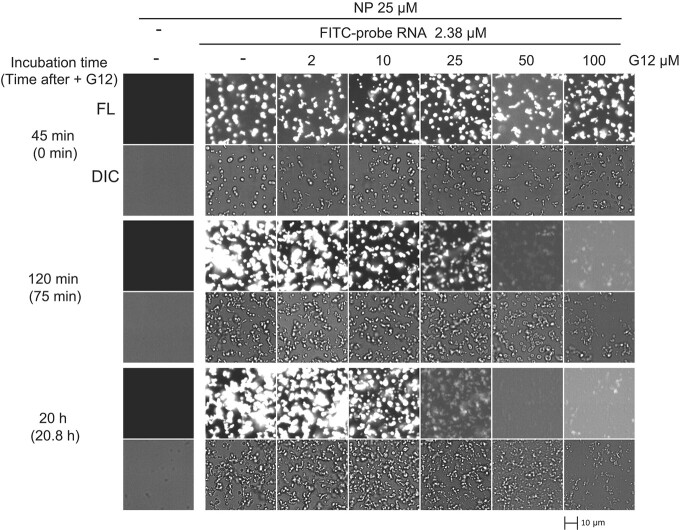
**The FITC-probe RNA in the NP filamentous/droplet-like structures was excluded by guanosine 12-mer (G12).** NP (25 μM) was mixed with 2.38 μM of FITC-labeled probe RNA for 45 min and further incubated with the indicated concentration of G12 oligomer for 75 min and 21 h. FITC-labeled and/or G12- controls are indicated as ‘-.’ DIC and fluorescent images were obtained at the indicated time. Time after the addition of probe RNA and, in parentheses, time after the addition of G12 are indicated. A scale bar of 10 μm is shown.

A previous report showed that hexameric RNAs formed 2:2 N protein/RNA complexes, whereas longer oligomeric RNAs exhibited lower dissociation constants and induced higher oligomerization of N proteins *(**21**)*. Given the strong effect of G6 on probe RNA-induced NP phase separation, we tested the inhibitory effect of a longer guanosine oligomeric RNA on N protein–RNA targeting. We examined the effect of a guanosine oligomer extended to a 12-mer (G12). As shown in Fig. 2C, G12 enhanced the filamentous aggregates observed with G6 (Fig. 2C). We next investigated whether G12 induced changes in probe-RNA-induced filamentous/droplet-like structures in a time- and concentration-dependent manner (Fig. 4). G12 was added after the formation of the FITC-probe RNA–induced structures of NP in a 45-min incubation. As shown in Fig. 4, the fluorescence of the FITC-labeled NP structures was strongly abated by G12 at concentrations of more than 25 μM. Instead, non-fluorescent filamentous/droplet structures were formed under those conditions. Therefore, G12 is thought to exclude and displace the FITC-probe RNA from the filamentous/droplet-like structures. A higher concentration (100 μM) of G12 inhibited the formation of the structures per se (Fig. 4).

**Fig. 5:**
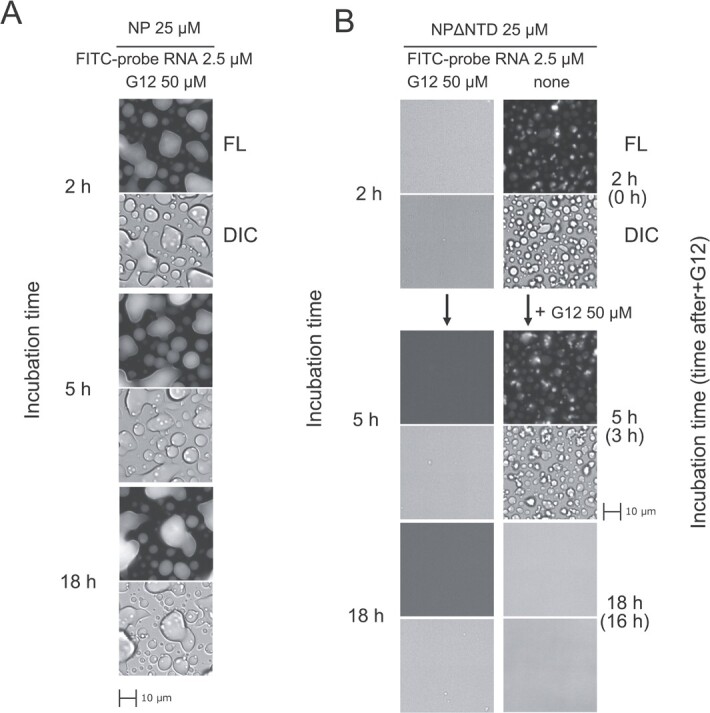
**The effect of the simultaneous addition of G12 and FITC-probe RNA on the phase separation of NP and NPΔNTD. A.** G12 and FITC-probe RNA were mixed with NP simultaneously. **B**. Similar to A, FITC-probe RNA was mixed with NPΔNTD with G12 simultaneously (left) or 2 h later (right). Fluorescent images (FL) and DIC images (DIC) were obtained after 2, 5 and 18 h.

Next, we investigated the effects of the simultaneous addition of G12 and the FITC-probe RNA to NP. As shown in Fig. 5A, LLPS-induced large droplets of NP were formed at equilibrium within 2 h, whereas the FITC-labeled droplet formation of NPΔNTD was completely inhibited in the presence of G12. In addition, the complete disruption of pre-existing droplets of NPΔNTD was observed within 18 h (Fig. 5B). These results demonstrated the strong inhibitory effect of G12 on the phase separation and probe RNA interaction of NP and NPΔNTD.

### Effects of modified guanosine oligomers on probe RNA-induced N protein filamentous/droplet-like structures

**Fig. 6:**
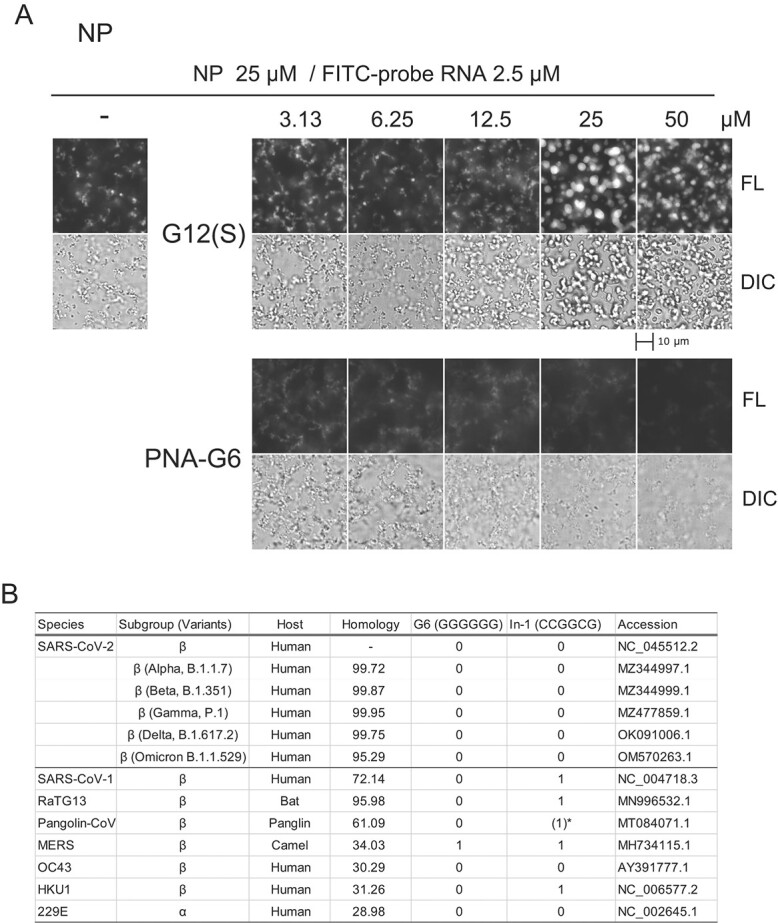
**The effects of phosphorothioate-modified guanosine 12-mer (G12(S)) and peptide nucleic acid guanosine hexamer (PNA-G6) on the phase separation of NP. A**. NP (25 μM) was incubated with FITC-probe RNA (2.5 μM) for 2 h, then further incubated with the indicated concentration of the oligomers for 16 h. Corresponding data for NPΔNTD are shown in Supplementary Fig. S4. B. In-1 and G6 are not found in SARS-CoV-2 variants or most other coronavirus species. Variants’ names are indicated by Pango lineage. The GenBank accession numbers of the used sequences are shown. The numbers of sequences corresponding to G6 and In-1 appearing in the positive and negative strands of each virus are shown. An asterisk indicates one In-1 sequence found in the negative strand.

In the drug discovery of nucleotide oligomers, phosphorothioate and protein nucleic acid (PNA) modifications are often applied to stabilize nucleotides *in vivo* and enhance their efficacy *(**25**)*. Therefore, we investigated the effect of modified guanosine oligomers on the small filamentous/droplet-like structures of NP. As shown in Fig. 6A, a phosphorothioate-modified guanosine 12-mer (G12(S)) inhibited the structures (at 6.25 μM) and coalesced at high concentrations (>25 μM). A similar concentration-dependent effect was observed for NPΔNTD (Supplementary Fig. S4). A peptide nucleic acid-guanosine hexamer (PNA-G6) strongly inhibited the formation of the small structures but did not affect NPΔNTD droplet formation (Supplementary Fig. S4). These results suggested that the modified guanosine oligomers also altered probe RNA-induced N protein aggregation.

## Discussion

Coronavirus has a long positive-stranded genome of ~30 kb. This is the largest genome compared to other RNA viruses, such as poliovirus (7.5 kb), influenza A (13.6 kb) and hepatitis C (10–11 kb). Although the ultrastructure of vRNP-containing virus particles of SARS-CoV-2 has recently been elucidated *(**5**,**11**)*, the interaction between genomic RNA and the N protein remains unclear. Understanding how genomic RNA interacts with the N protein and whether genomic RNA binds to the N protein in a sequence-specific manner for vRNA formation is important to identify new antiviral drug targets.

We focused on sequence specificity regarding binding to the NTD and the induction and inhibition of phase separation. We identified two hexameric sequences (G6 and In-1) whose sequences are not found in the SARS-CoV-2 genome or its negative-strand sequences. We noted that the two hexamers are absent in the genome sequences of SARS-CoV-2 variants (Alpha, Beta, Gamma, Delta and Omicron) as well as other human coronaviruses (229E and OC43; Fig. 6B). As the 3D structures of the NTD, CTD and IDRs are well conserved *(**8**)*, the mechanisms of vRNP formation may also be conserved, suggesting the importance of the genetic exclusion of these hexameric sequences.

The N protein can undergo spherical droplet formation through LLPS, in which multiple N proteins and multiple RNAs undergo protein–protein and protein–RNA interactions. However, it is believed that one coronaviral genome binds to the N protein to form a vRNP, packaging the virus *(**5**,**8**,**11**)*. Therefore, we considered that the phenomenon wherein droplets do not expand randomly is an intrinsic function of the N protein.

Our results suggest that the NTD may play an important role in the function of the N protein. First, the size and FITC-probe intensity of droplets increased with the loss of the NTD (Fig. 3 and Supplementary Fig. S3, the first panel for each dataset). Second, the FITC-probe RNA–induced droplets in the presence of G6 and In-1 enhanced the small filamentous/droplet-like structures of NP, and the loss of the NTD caused the droplets to undergo coalescence and phase separation with unclear phase boundaries (Fig. 3 and Supplementary Fig. S3). Third, the FITC-probe RNA–induced NPΔNTD droplets were completely disrupted by G12 (Fig. 5B), whereas in the case of NP, the small structures of NP remained intact with G12 and the FITC-probe RNA was expelled (Fig. 4). These results suggested that RNA-bound NTD is required for the formation and stabilization of the small filamentous/droplet-like structures of N protein and probe RNA.

Our results also showed that the NTD and non-NTD of the N protein exhibit sequence-specific interactions with oligomeric RNA. There was a difference in activity between the single nucleotide substitution variants In-1 and mIn-1. We found that mIn-1’s inhibitory effect on the NTD–RNA interaction was stronger than that of In-1 (Fig. 1C and D). Furthermore, In-1, but not mIn-1, induced large spherical droplets of NP (Fig. 2B) and NPΔNTD (Fig. 3, NPΔNTD). These results suggest that In-1 may interact with the non-NTD regions of the N protein. Meanwhile, G6 may interact with the NTD to inhibit NTD–RNA binding (Fig. 1C and D) and may also interact with the non-NTD regions to induce the coalescence of droplets (Fig. 3, NPΔNTD). Therefore, our results suggest that several regions of the N protein each have different RNA sequence binding specificities.

Small molecules that reduced RNA–N protein droplets were identified *(**14**,**17**,**20**)*. Zhao et al. identified a poly ADP-ribose polymerase (PARP) inhibitor, CL218, which could bind to the SARS-CoV-2 N protein to elevate the size of droplets and enhance the anti-SARS-CoV-2 effect of remdesivir by less than 2-fold *(**20**)*. Iserman et al. *(**14**)* demonstrated that the size of N protein–driven droplets was reduced in the presence of either lipoic acid, which dissolves cellular stress granules, or kanamycin, previously shown to electrostatically bind to nucleic acids *(**26**)*. Other groups screened approved drugs in terms of their effect on LLPS droplet formation and identified nelfinavir mesylate, which slightly altered droplet shape and inhibited 10% of SARS-CoV-2-induced cytopathic effects on infected cells *(**17**)*. The fact that these compounds exhibited weak alterations in N protein droplets and some of these compounds slightly reduced viral propagation supports the efficacy of the N protein droplet inhibition assay as an antiviral screening system *(**14**,**17**,**20**)*.

We demonstrated that G12 could expel pre-existing FITC-probe RNA from the small filamentous/droplet-like structures of NP (Fig. 4) and inhibit their formation in the simultaneous presence of both probe RNA and G12 of NP (Fig. 5). G12 may act as a multivalent binder, that is, one G12 molecule may bind to multiple NTD and/or non-NTD regions simultaneously, thus exhibiting stronger inhibitory activity than G6.

To enhance the bioavailability of oligonucleotide drugs, the phosphodiester bond and ribose structure are often modified *(**25**)*. Although the effects of the phosphorothioate-modified guanosine 12-mer (G12(S)) and the peptide nucleic acid-guanosine hexamer (PNA-G6) differed from that of G12, they did affect N protein-induced structures (Fig. 6A). Further modification of the phosphodiester bond, ribose components and length may enhance the inhibitory effect of G12 on the N protein–probe RNA interaction.

The potent inhibitory activity of G12 and its derivatives on the N protein–RNA interaction suggests that G12 and its derivatives may be potential antiviral agents for SARS-CoV-2 if their effects strongly exceed putative cytotoxicity. We are currently looking into this possibility.

### Study limitations

We used only a 32-base sequence, which is homologous to several *β*-coronaviruses (Supplementary Fig. S1C), as probe RNA in this study. The probe sequence did not include ISs, the high-affinity region for N protein binding *(**14**)*. There is no information based on similar experiments using other sequences and different lengths of genomic regions as probes. Thus, the appearance of SARS-CoV-2 N protein-driven LLPS or droplets may be different when IS-containing sequences or other regions are used. However, using our experimental systems as comparative assays, here, we provide evidence for possible NTD function and the sequence-specific inhibitory effects of the oligomeric RNAs on the RNA–N protein complex.

## Materials and Methods

### Reagents

Oligonucleotides (RNA and DNA), phosphorothioate-modified guanosine 12-mer, 32-base probe RNA and FITC-labeled 32-base probe RNA were purchased from Fasmac (Kanagawa, Japan). The 32-base probe sequence was derived from NC_045512.2 (nt29733–29764: CGAGGCCACGCGGAGUACGAUCGAGUGUACAG). The guanosine 12-mer was purchased from Aji Bio-Pharma (Osaka, Japan). PNA-G6 was synthesized using Biotage Initiator+ microwave peptide synthesizer (Biotage, Uppsala, Sweden) based on Fmoc/Bhoc chemistry with a Rink-Amide-Chem Matrix resin (Biotage). The crude product was purified and verified according to the published literature *(**27**)*. DNA oligonucleotides were purchased from Fasmac. Other reagents were purchased from Nacalai Tesque (Kyoto, Japan).

### Expression and purification of full-length NTD and NPΔNTD SARS-CoV-2 N proteins from *E. coli*

The NTD region (aa 46–174) of SARS-CoV-2 N protein cDNA was synthesized (Eurofins Genomics, Tokyo, Japan) and cloned between the *Nde*I and *Bam*HI sites of pET-15b (Novagen, Merck, Darmstadt, Germany) using restriction enzymes or the In-Fusion reaction (Takara bio, Shiga, Japan). Similarly, the full-length SARS-CoV-2 N protein was PCR-amplified from the DNA clone pCMV3-2019-nCoV-NP (Sino Biological, Hong Kong, PRC). To construct NPΔNTD, aa 46 to 174 of the N protein were deleted by PCR. *E. coli* competent cells of BL21-CodonPlus (DE3)-RIL (Agilent, California, USA) were transformed with the resulting plasmids. The expression of the HIS-tag proteins (NP, NPΔNTD and NTD) was induced in the presence of 1 mM isopropyl β-D-1-thiogalactopyranoside in 400 ml LB (OD_600_ = 0.4) at 25°C for 6 h. For NTD purification, *E. coli* lysates were prepared by sonication in 10 ml Buffer A (phosphate buffered saline [PBS], 20% glycerol, 0.1% Tween 20, 10 mM imidazole and 1 mg/ml lysozyme) and centrifugation (13,400 g for 30 min at 4°C). The supernatants were mixed with 0.8 ml Ni-NTA agarose (Qiagen, Venlo, Netherlands) for 3 h at 4°C. The Ni-NTA agarose was washed with Buffer B (PBS, 20% glycerol and 20 mM imidazole) and eluted with 2 ml Buffer C (PBS, 20% glycerol, 0.1% Tween 20 and 250 mM imidazole). The eluate was purified again with Ni-NTA agarose. The eluate was dialysed with PBS overnight at 4°C.

For NP and NPΔNTD purification, a denaturing condition was used to prevent the co-purification of endogenous *E. coli* RNA, as described by Carlson et al. *(**12**)*. *E. coli* pellets were sonicated in Buffer Ad (50 mM 4-(2-hydroxyethyl)-1-piperazineethanesulfonic acid [HEPES] pH 7.5, 500 mM NaCl, 10% glycerol, 20 mM imidazole and 6 M urea). The purification steps were performed as described above, except that Buffer Ad was used for washing and Buffer Bd (50 mM HEPES pH 7.5, 500 mM NaCl, 10% glycerol, 250 mM imidazole and 6 M urea) for elution. NP and NPΔNTD were renatured by dialysis in 1 l Buffer Cn (50 mM HEPES pH 7.5, 50 mM NaCl and 10% glycerol) for more than 12 h. The dialysis step was repeated.

### Preparation of ^32^P-labeled probe RNA

Synthetic DNA of the T7 promoter followed by 32 bases of the SARS-CoV-2 3′-noncoding region (NC_045512.2, nt29733–29764: TAATACGACTCACTATAGGGcgaggccacgcggagtacgatcgagtgtacag) was used as a template for 32-base RNA probe synthesis. A MEGAscript T7 Transcription Kit (Ambion, ThermoFisher, MA, USA) was used to synthesize the 32-base RNA probe. A volume of 0.2 μl each of GTP, ATP and UTP, 0.05 μl of CTP and 1 μl of α-^32^P-CTP (370 MBq/ml, 29.6 TBq/mmol, PerkinElmer NEG008X) were mixed with 1 μg of template DNA and 2 μl of enzyme mix in 20 μl of reaction mixture, and the resulting mixture was incubated for 2 h at 37°C. After TURBO DNase (ThermoFisher) treatment, ^32^P-labeled RNA (^32^P-RNA) was purified using QIAamp viral RNA mini (Qiagen, Venlo, Netherlands) according to the manufacturer’s instructions without using carrier RNA.

### Electrophoretic mobility shift assay (EMSA)

EMSA was performed generally as described previously *(**23**)*. First, 5 ng of ^32^P-RNA was incubated with 800 ng NTD and inhibitor in 10 μl of 1 U/μl RNase inhibitor, 10 mM HEPES, and 50 mM NaCl at pH 7.5 for 20 min. The samples were separated by 2% agarose gel electrophoresis (100 V, 40 min). Then, the gel was fixed with 10% acetate and 10% methanol and dried for 40 min with a gel dryer (Bio Craft, Tokyo, Japan). Images of the ^32^P band were detected using an imaging plate (IP, Fujifilm, Tokyo, Japan) and an IP scanner (FLA3000F, Fujifilm).

### Phase separation assay

Phase separation was observed in an mPEG silane-coated glass-bottomed 384-well plate (SensoPlate, Greiner Bio-One, Kremsmünster, Austria). Plate treatment and mPEG Silane 5 K (Biopharma PEG Scientific, MA, USA) coating were performed as described previously *(**24**)*. Each indicated concentration of NP or NPΔNTD, probe RNA (unlabeled or a mixture of unlabeled and FITC-labeled) and inhibitor oligomer was mixed in 50 mM HEPES/Na (pH 7.5), 10% glycerol, 50 mM NaCl and 1 U/μl RNasin at 27°C. DIC and fluorescent microscopic images were obtained at the indicated time points using a DIC-installed Leica DMR with a 40× objective (HCX PLAPD 40×/0.75 U-V-I) and a GFP filter for FITC equipped with Zyla 4.2P sCMOS (Andor Technology, Belfast, UK). Images were acquired as stack files using μ-manager under the same conditions of excitation and CCD camera sensitivity as for capturing fluorescence images. The stack files were separated after adjusting the contrast and brightness of each stack file simultaneously using ImageJ. Steady-state levels and shapes of the droplets appeared after overnight incubation (>12~16 h).

### Coronavirus genome analysis

The homology of genome sequences between SARS-CoV-2 and other coronavirus strains and variants was determined using CLUSTALW from Kyoto University’s Bioinformatics Center (https://www.genome.jp/tools-bin/clustalw). The selected variants of SARS-CoV-2 correspond to variants of concern (VOC) indicated by the WHO.
